# Cerebral Hemodynamics and Carotid Atherosclerosis in Patients With Subcortical Ischemic Vascular Dementia

**DOI:** 10.3389/fnagi.2021.741881

**Published:** 2021-11-22

**Authors:** Xiao-Jiao Liu, Ping Che, Mengya Xing, Xiao-Bing Tian, Chunli Gao, Xiuyan Li, Nan Zhang

**Affiliations:** ^1^Department of Neurology, Tianjin Medical University General Hospital Airport Site, Tianjin, China; ^2^Department of Neurology, Tianjin Neurological Institute, Tianjin Medical University General Hospital, Tianjin, China

**Keywords:** subcortical ischemic vascular dementia, Alzheimer’s disease, transcranial Doppler, mean flow velocity, pulsatility index

## Abstract

A growing body of evidence indicates that atherosclerosis is correlated with cerebral small vessel disease and contributes to cognitive decline. This study aimed to explore the characteristics and contributions of intracranial hemodynamics and carotid atherosclerosis to cognitive dysfunction in subjects with subcortical ischemic vascular dementia (SIVD). Notably, 44 patients with SIVD, 30 patients with Alzheimer’s disease (AD), and 30 healthy controls (HCs) were recruited from our longitudinal MRI study for AD and SIVD (ChiCTR1900027943). The cerebral mean flow velocity (MFV) and pulsatility index (PI) of both anterior and posterior circulations, artery plaque, and lumen diameter in carotid arteries were investigated using transcranial Doppler and carotid ultrasound, respectively. Their correlations with cognitive function were analyzed in patients with dementia. Decreased MFV and increased PI were found in patients with SIVD and AD. Patients with SIVD showed lower MFV and higher PI in the bilateral posterior cerebral arteries compared to patients with AD. Increases in lumen diameter, number of arteries with plaque, and total carotid plaque score were found in patients with SIVD. The Mini-Mental State Examination score was positively correlated with the MFV and negatively correlated with the PI of most major cerebral arteries, while it was negatively correlated with the lumen diameter of the common carotid artery, number of arteries with plaque, and total carotid plaque score in patients with dementia. There were also correlations between these parameters of some arteries and memory and executive function. Our results provide additional evidence suggesting that the pathological changes in macrovascular structure and function are correlated with cognitive impairment in dementia patients with SIVD and to a lesser extent AD.

## Introduction

Vascular dementia (VaD) is the second most commonly diagnosed type of dementia after Alzheimer’s disease (AD) in people over the age of 70 years. According to the Vascular Impairment of Cognition Classification Consensus Study, VaD can be categorized into poststroke dementia, subcortical ischemic vascular dementia (SIVD), multi-infarct (cortical) dementia, and mixed dementia (Skrobot et al., 2018). SIVD is a clinically homogeneous subtype of VaD, typically characterized by cognitive impairment, mental and mood-behavioral disorders, gait instability, and incontinence. Approximately 36–67% of patients with VaD exhibit the pathological changes of cerebral small vascular disease, which is the main pathogenesis of SIVD (Roman et al., 2002).

Brain imaging features of patients with SIVD are characterized by white matter hyperintensities (WMH), lacunes, subcortical infarcts, enlarged perivascular spaces (PVS), microbleeds, and brain atrophy (Moretti and Caruso, 2020). Recent studies revealed correlations between hemodynamic and structural changes in large vessels and SIVD imaging features using transcranial Doppler (TCD) and carotid ultrasound. In a community-based study, elevated pulsatility index (PI) of the middle cerebral artery (MCA), increased plaque number, and enlarged diameter of carotid arteries are correlated with more lacunes and larger WMH volume (Mok et al., 2012). Cerebral perfusion and vascular resistance measured by TCD are significantly associated with WMH severity and executive function in non-demented elderly subjects (Vinciguerra et al., 2019). Moreover, PI of the MCA is significantly correlated with the scores on the Mini-Mental State Examination (MMSE), Trail Making Test-A (TMT-A), and TMT-B in patients with lacunar infarction (Altmann et al., 2016).

A large amount of clinical and postmortem evidence has revealed structural and functional changes in cerebral large vessels in patients with AD. Previous studies reported that patients with AD, in particular, apolipoprotein E (APOE) ε4 genotype carriers, have lower mean flow velocity (MFV) and higher PI of the MCA compared with age-matched non-demented individuals (Roher et al., 2011). A systematic review concluded that patients with mild cognitive impairment (MCI) show decreased cerebral blood flow velocity in the bilateral anterior cerebral artery (ACA) and MCA (especially among APOE ε4 carriers) compared with healthy subjects. This hemodynamic shift in patients with MCI also predicts their conversion to AD (Beishon et al., 2017). In addition, cerebral atherosclerotic stenosis is more common in patients with neuropathologically confirmed AD than non-demented individuals (Roher et al., 2004).

Major risk factors of arteriolosclerosis in small vessel diseases, such as aging, hypertension, diabetes, smoking, hyperhomocysteinemia, obesity, and dyslipidemia, also contribute to arteriosclerosis in large arteries. Moreover, cerebral hemodynamic changes indirectly accelerate cerebral edema, inter-arterial space dilation, and interstitial fluid retention. These changes are subsequently reflected on MRI, indicating the characteristics of SIVD. However, the pathological and functional changes in large vessels and their associations with cognitive function in patients with SIVD are still unknown. In this study, we investigated both anterior and posterior intracranial hemodynamics measured by TCD and carotid artery changes measured by carotid ultrasound in patients with SIVD. We compared the results to those in patients with AD, whose diagnosis was supported by fluorodeoxyglucose (FDG) and amyloid PET scans. Our results underscore the potential contributions of cerebral and carotid atherosclerosis to cognitive impairment in patients with SIVD and AD.

## Materials and Methods

### Participants

A total of 104 participants, including 44 patients with SIVD, 30 patients with AD, and 30 age- and sex-matched healthy controls (HCs), were recruited from our longitudinal MRI study for AD and SIVD (ChiCTR1900027943). The diagnoses of AD and SIVD were supported by FDG and amyloid PET and multimodal MRI, respectively. All participants (aged from 50 to 85 years) underwent systematic evaluations, including medical history collection, physical and neurological examinations, neuropsychological assessment, laboratory tests, and brain MRI. The study was approved by the Ethics Committee of the Tianjin Medical University General Hospital (IRB2017-063-01). Written consent forms were signed by all participants and their legal guardians.

The diagnostic criteria for patients with dementia met the criteria for the major neurocognitive disorder (Sachdev et al., 2015) according to the fifth edition of the *Diagnostic and Statistical Manual of Mental Disorders*. They also had MMSE scores within the range of 10–26, and the Clinical Dementia Rating (CDR) scores within 0.5–2. Patients with SIVD met the diagnostic criteria for VaD according to the *Vascular Behavioral and Cognitive Disorders* (Sachdev et al., 2014). Patients with AD met the research diagnostic criteria for typical AD of International Working Group-2 (Dubois et al., 2014), presented with early and significant episodic memory impairment, and had positive results on ^11^C-labeled Pittsburgh compound B PET. Patients with cognitive impairment caused by other neurological diseases, mental disorders, and medical conditions, such as frontotemporal lobar degeneration, dementia with Lewy bodies, Parkinson’s disease, multiple sclerosis, hydrocephalus, severe depression, schizophrenia, thyroid dysfunction, vitamin B12 deficiency, and syphilis or human immunodeficiency virus infections, were excluded. All HCs demonstrated normal performances on neuropsychological assessments (MMSE score > 26 and CDR score of 0) with no subjective cognitive decline.

The MRI criteria were also applied for each group. Patients with SIVD were characterized by the following: (1) multiple (≥3) supratentorial subcortical lacunes (3–20 mm in diameter) with/without WMH of any degree; or (2) moderate-to-severe WMH in either periventricular region or deep white matter according to the Fazekas rating scale (score ≥ 2 with/without lacunes) (Fazekas et al., 1987); or (3) strategically located subcortical small infarcts in the deep gray matter; and (4) no obvious hippocampal atrophy (Scheltens’ medial temporal lobe atrophy scale < 2 for age ≤ 75 years old or < 3 for age > 75 years old) indicating the possibility of comorbid AD (Scheltens et al., 1992). Patients with AD were excluded by evidence of significant cerebrovascular diseases such as multiple cortical infarction or strategic infarction, >1 lacune in the basal ganglia, or severe WMH. There was no clinically significant atrophy in the medial temporal lobe or other brain areas or cerebrovascular diseases shown on MRI of HC.

All participants had no history of drug or alcohol abuse, no severe depression (Hamilton Depression Scale score < 18) (Hamilton, 1967), and no severe visual or auditory disability that could influence cognitive assessment performance. The doses of medication that might affect the cognitive function or cerebral hemodynamics were stable within 4 weeks before neuropsychological assessment and ultrasonography measurement.

### Ultrasonography Measurement

The intracranial hemodynamics were measured by TCD (Delikai TC9-NB, Guangdong, China). During TCD assessment, all participants adopted a supine position in a quiet and warm indoor environment. The resting-state parameters of peak systolic blood flow velocity (PSV), end diastolic blood flow velocity (EDV), and MFV, which partly represent arterial stenosis and cerebral blood flow, were obtained through the bilateral temporal and occipital windows. PI, equivalent to pulse pressure, was used as an indicator to identify distal blood flow resistance and vessel wall elasticity and was calculated by the formula of (PSV−EDV)/MFV. We analyzed the MFV and PI of the bilateral intracranial carotid arteries (ICAs), ACAs, MCAs, posterior cerebral arteries (PCAs) and vertebral arteries (VAs), and basilar artery (BA), including the proximal BA (pBA) and distal BA (dBA).

Carotid artery plaque and lumen diameter were evaluated through high-resolution B-mode ultrasound (Hitachi HI VISION, Shenzhen, China) examination with a 7.5-MHz probe. The atherosclerotic plaque was measured at six carotid segments, namely, the left and right common carotid artery (CCA), carotid bifurcation, and extracranial internal carotid artery (EICA). The plaque was defined as focal structures that invaded the arterial lumen at least 0.5 mm or with thickness >1.5 mm measured from the media-adventitia interface to the intima-lumen interface (Touboul et al., 2007) and was evaluated at each carotid artery segment. Apart from the number of plaque and the number of arteries with plaque, the carotid plaque score (Ihle-Hansen et al., 2019) was also used to quantify plaque in this study. The largest plaque in each section was scored according to its diameter (≥1.5, ≥2.5, and ≥3.5 mm were scored as 1, 2, and 3 points, respectively), and the total score of each segment was calculated and ranged from 0 to 18. Compared with the measurements of total plaque area and diameter, the plaque score was easier to evaluate and had better inter-operator reliability. The lumen diameters of the bilateral CCA, EICA, and VA during the diastole of each cardiac cycle were measured as the average of the distance between the leading edges of far- and near-wall lumen-intima interfaces (Brisset et al., 2013).

### Neuropsychological Assessment

All participants underwent neuropsychological assessments. Global cognitive impairment was measured by the MMSE. Memory and executive function (i.e., the main cognitive functions impaired in patients with AD and SIVD, respectively) were also analyzed. The memory was calculated by the average *Z*-scores of the total learning, delayed recall, and recognition in the Rey Auditory Verbal Learning Test and the Brief Visuospatial Memory Test-Revised. The executive function was calculated by the average *Z*-scores of the TMT-B and the Stroop Color and Word Test. *Z*-scores were converted using the means and SDs of the HC group. All the cognitive data were collected within 1 week before or after the ultrasonographic measurements.

### Statistical Analysis

All the data were analyzed by SPSS version 22.0 (IBM Corp. Armonk, NY, United States). The values are provided as a number or a percentage for categorical variables, mean ± SD for continuous variables, and 95% CIs for multiple logistic regression models. The differences in clinical characteristics and TCD and the carotid ultrasound parameters between patients with SIVD, patients with AD, and HCs were compared using the one-way ANOVAs for continuous variables or the Chi-square tests for categorical variables. The multiple logistic regression analysis was used for the correlation analysis of TCD and carotid ultrasound parameters with global cognition, memory, and executive function in patients with dementia (i.e., SIVD and AD). Age, sex, education, and vascular risk factors were adjusted during the regression analysis. *P* < 0.05 was considered statistically significant.

## Results

### Demographic Characteristics

The demographic characteristics of all subjects are shown in Table 1. In total, 44 patients with SIVD (mean age of 71.6 years, median age of 72 years, 41.2% females), 30 patients with AD (mean age of 68.7 years, median age of 68 years, 70.3% females), and 30 HCs (mean age of 66.2 years, median age of 66 years, 63.3% females) were included in this study. All participants were right-handed. No statistically significant differences were found in age, educational level, and body mass index (BMI) among the three groups. However, the HC group was slightly younger than the SIVD and AD groups, but the differences were not statistically significant. Although all participants were between 50 and 85 years old, the age distribution was slightly different across three groups (SIVD vs. AD vs. HC: 50–59 years, 4.55 vs. 16.67 vs. 26.67%; 60–79 years, 84.09 vs. 73.33 vs. 70%; and 80–85 years, 11.36 vs. 10 vs. 3.33%). In addition, compared with the AD group, there was a lower proportion of females in the SIVD group. In terms of vascular risk factors, significantly higher incidences of stroke history (56.8 vs. 0 vs. 0%) and hypertension (72.7 vs. 26.7 vs. 46.7%) were found in patients with SIVD than in the AD and HC groups. A significantly higher proportion of diabetes mellitus (40.9 vs. 3.3%) was found in patients with SIVD compared to those with AD. A significantly lower incidence of hypercholesterolemia (30 vs. 60%) was found in patients with AD compared to HCs. No participants had comorbid chronic obstructive pulmonary disease (COPD). More patients with SIVD were using statins as compared to patients with AD and HCs (36.4 vs. 13.3 vs. 20.0%), but this difference was not significant. The SIVD and AD groups showed significantly lower MMSE score (20.4 ± 3.22 vs. 19.6 ± 2.44 vs. 28.2 ± 1.20) and lower *Z*-scores in memory assessments (−2.11 ± 0.87 vs. −2.90 ± 0.87 vs. 0.08 ± 0.57) and executive function tests (−2.46 ± 0.98 vs. −2.10 ± 0.98 vs. 0.02 ± 0.61) than the HC group. No significant difference in the MMSE score was found between the SIVD and AD groups. However, patients with SIVD had better performance in memory and worse performance in executive function than patients with AD.

**TABLE 1:** Group characteristics.

	**SIVD (*n* = 44)**	**AD (*n* = 30)**	**HC (*n* = 30)**	**F/χ^2^**
Age, years	71.6 (7.90)	68.7 (8.81)	66.2 (6.29)	F = 4.81
Sex, female/male*	18/26	21/9	19/11	χ^2^ = 7.10
Education, years	10.2 (2.57)	10.4 (4.48)	11.3 (3.37)	F = 0.33
BMI	24.3 (2.69)	24.2 (2.15)	24.5 (2.64)	F = 0.66
Hypercholesterolemia^†^, %	45.5	30.0	60.0	χ^2^ = 5.45
Hypertension*^‡^, %	72.7	26.7	46.7	χ^2^ = 15.63
Diabetes mellitus*, %	40.9	3.3	20.0	χ^2^ = 14.17
Stroke, %	56.8	0	0	–
Coronary artery disease, %	22.7	6.7	6.7	χ^2^ = 5.62
COPD, %	0	0	0	–
Statin use, %	36.4	13.3	20.0	χ^2^ = 5.61
MMSE^†⁣‡^	20.4 (3.22)	19.6 (2.44)	28.2 (1.20)	F = 129.24
Memory*^†⁣‡^	−2.11 (0.87)	−2.90 (0.87)	0.08 (0.57)	F = 115.32
Executive function*^†⁣‡^	−2.46 (0.98)	−2.10 (0.98)	0.02 (0.61)	F = 75.01

*Values are provided as mean (SD) unless otherwise indicated. Memory and executive function were analyzed using composite *Z*-scores.*

*AD, Alzheimer’s disease; BMI, body mass index; COPD, chronic obstructive pulmonary disease; HC, healthy control; MMSE, Mini-Mental State Examination; SIVD, subcortical ischemic vascular dementia.*

*SIVD vs. AD, *P* < 0.05.

*^‡^SIVD vs. HC, *P* < 0.05.*

*^†^AD vs. HC, *P* < 0.05.*

### Mean Flow Velocity Features

Significant differences in the MFV of the bilateral ICAs, PCAs, and VAs; right ACA and MCA; and pBA and dBA were found among the three groups (Table 2). Compared to the HC group, both patients with SIVD and AD showed lower MFVs of the bilateral PCAs; right ICA, ACA, MCA, and VA; and pBA and dBA. Patients with SIVD also showed lower MFVs of the left ICA and VA than HCs, as well as lower MFV of the left PCA than patients with AD.

**TABLE 2:** Mean flow velocities (MFVs) of insonated arteries by the group.

	**SIVD (*n* = 44)**	**AD (*n* = 30)**	**HC (*n* = 30)**	**F**
R ICA^†⁣‡^	57.02 (13.09)	53.57 (8.99)	62.77 (10.44)	5.11
R ACA^†⁣‡^	46.77 (9.93)	47.93 (7.43)	53.73 (9.93)	5.35
R MCA^†⁣‡^	46.14 (5.20)	45.83 (4.72)	57.10 (10.92)	25.18
R PCA^†⁣‡^	31.45 (6.66)	32.63 (5.49)	36.83 (7.80)	5.97
R VA^†⁣‡^	28.30 (5.72)	30.43 (4.58)	44.10 (5.58)	83.93
pBA^†⁣‡^	27.84 (5.94)	27.70 (5.13)	39.60 (6.93)	41.00
dBA^†⁣‡^	23.32 (5.50)	25.60 (4.99)	35.83 (7.77)	45.87
L ICA^‡^	52.41 (13.06)	55.37 (11.25)	60.67 (12.19)	4.02
L ACA	48.11 (12.05)	50.87 (15.54)	53.13 (13.66)	1.25
L MCA	55.68 (12.65)	56.23 (7.04)	59.07 (8.65)	1.05
L PCA*^†⁣‡^	29.09 (7.68)	36.73 (11.47)	42.90 (5.96)	23.89
L VA^‡^	25.61 (7.07)	28.47 (5.18)	29.53 (9.17)	2.90

*Values (cm/s) are provided as mean (SD).*

*ACA, anterior cerebral artery; AD, Alzheimer’s disease; dBA, distal basilar artery; HC, healthy control; ICA, intracranial internal carotid artery; L, left; MCA, middle cerebral artery; MFV, mean flow velocity; pBA, proximal basilar artery; PCA, posterior cerebral artery; R, right; SIVD, subcortical ischemic vascular dementia; VA, vertebral artery.*

*SIVD vs. AD, *P* < 0.05.

*^‡^SIVD vs. HC, *P* < 0.05.*

*^†^AD vs. HC, *P* < 0.05.*

### Pulsatility Index Features

The PI of most measured arteries was significantly higher in patients with SIVD than in HCs, including the bilateral ACAs, MCAs, and PCAs; right ICA and VA; and pBA and dBA (Table 3). Patients with AD also demonstrated increased PI of the bilateral MCAs, left ACA, as well as pBA and dBA compared to HCs. Patients with SIVD had higher PI of the bilateral PCAs than patients with AD; meanwhile, patients with AD had higher PI of the pBA and dBA than patients with SIVD.

**TABLE 3:** Pulsatility indices (PIs) of insonated arteries by the group.

	**SIVD (*n* = 44)**	**AD (*n* = 30)**	**HC (*n* = 30)**	**F**
R ICA^‡^	1.00 (0.15)	0.97 (0.18)	0.91 (0.84)	3.45
R ACA^‡^	1.02 (0.15)	0.97 (0.19)	0.92 (0.13)	3.51
R MCA^†⁣‡^	1.19 (0.16)	1.18 (0.12)	0.88 (0.14)	11.67
R PCA*^‡^	1.03 (0.14)	0.94 (0.21)	0.91 (0.09)	6.40
R VA^‡^	1.02 (0.15)	0.98 (0.14)	0.93 (0.08)	4.84
pBA*^†⁣‡^	0.98 (0.13)	1.11 (0.16)	0.85 (0.05)	31.75
dBA*^†⁣‡^	0.94 (0.13)	1.06 (0.14)	0.82 (0.07)	28.43
L ICA	1.03 (0.14)	0.96 (0.20)	0.95 (0.10)	2.97
L ACA^†⁣‡^	1.03 (0.13)	1.02 (0.20)	0.94 (0.10)	4.95
L MCA^†⁣‡^	1.05 (0.14)	1.00 (0.12)	0.87 (0.16)	15.15
L PCA*^‡^	1.03 (0.14)	0.93 (0.21)	0.92 (0.10)	5.87
L VA	1.00 (0.14)	1.00 (0.14)	0.94 (0.10)	2.43

*Values are provided as mean (SD).*

*ACA, anterior cerebral artery; AD, Alzheimer’s disease; dBA, distal basilar artery; HC, healthy control; ICA, intracranial internal carotid artery; L, left; MCA, middle cerebral artery; pBA, proximal basilar artery; PCA, posterior cerebral artery; PI, pulsatility index; R, right; SIVD, subcortical ischemic vascular dementia; VA, vertebral artery.*

*SIVD vs. AD, *P* < 0.05.

*^‡^SIVD vs. HC, *P* < 0.05.*

*^†^AD vs. HC, *P* < 0.05.*

### Lumen Diameter

**FIGURE 1:**
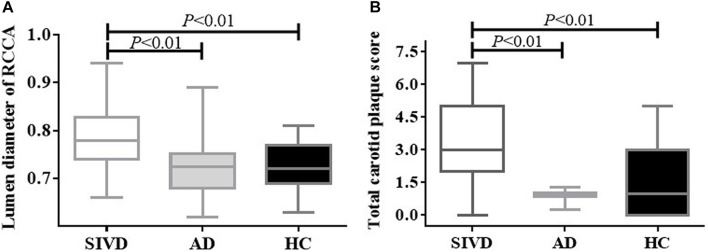
Lumen diameter of the right CCA and total carotid plaque score by the group. Patients with SIVD showed significantly increased lumen diameter **(A)** and total carotid plaque score **(B)** compared with the AD and HC groups (*P* < 0.01). There was no difference between the AD and HC groups (*P* > 0.05). AD, Alzheimer’s disease; CCA, common carotid artery; HC, healthy control; SIVD, subcortical ischemic vascular dementia.

Patients with SIVD showed significantly increased lumen diameters in the bilateral CCAs and left EICA compared to the AD and HC groups (Table 4 and Figure 1A). No difference in lumen diameter of any measured artery was found between patients with AD and HCs.

**TABLE 4:** Lumen diameter of carotid arteries by the group.

	**SIVD (*n* = 44)**	**AD (*n* = 30)**	**HC (*n* = 30)**	**F**
R CCA*^‡^	0.79 (0.06)	0.72 (0.06)	0.73 (0.06)	14.54
R EICA	0.49 (0.03)	0.48 (0.02)	0.48 (0.02)	2.90
R VA	0.34 (0.05)	0.32 (0.02)	0.33 (0.03)	2.00
L CCA*^‡^	0.78 (0.06)	0.73 (0.07)	0.72 (0.06)	11.20
L EICA*^‡^	0.49 (0.03)	0.48 (0.03)	0.48 (0.02)	3.40
L VA	0.36 (0.05)	0.35 (0.04)	0.33 (0.04)	3.03

*Values (cm) are provided as mean (SD).*

*AD, Alzheimer’s disease; CCA, common carotid artery; EICA, extracranial internal carotid artery; HC, healthy control; L, left; R, right; SIVD, subcortical ischemic vascular dementia; VA, vertebral artery.*

*SIVD vs. AD, *P* < 0.05.

*^‡^SIVD vs. HC, *P* < 0.05.*

*^†^AD vs. HC, *P* < 0.05.*

### Carotid Plaque Characteristics

Patients with SIVD showed significantly higher plaque number, number of arteries with plaque, and total carotid plaque score compared to HCs, as well as higher total carotid plaque score compared to patients with AD (Table 5 and Figure 1B). An increasing trend in the index for the carotid plaque was found in patients with AD compared to HCs, but this was not statistically significant.

**TABLE 5:** Carotid plaque by the group.

	**SIVD (*n* = 44)**	**AD (*n* = 30)**	**HC (*n* = 30)**	**F**
Plaque numbers^‡^	2.36 (1.22)	1.77 (1.48)	1.40 (1.54)	4.49
Number of arteries with plaque^‡^	2.16 (1.12)	1.60 (1.30)	1.23 (1.30)	5.30
Total carotid plaque score*^‡^	3.23 (1.78)	2.00 (1.68)	1.60 (1.71)	9.02

*Values are provided as mean (SD). Carotid arteries were divided into three segments on both sides (common carotid artery, bifurcation, and internal carotid artery), and plaques were quantified in each segment. The largest plaque in each segment was scored as 1, 2, or 3 according to its diameter (≥1.5, ≥2.5, and ≥3.5 mm, respectively). The total carotid plaque score was calculated as the sum of the scores for each segment, ranging from a minimum of 0 points to a maximum of 18 points.*

*AD, Alzheimer’s disease; HC, healthy control; SIVD, subcortical ischemic vascular dementia.*

*SIVD vs. AD, *P* < 0.05.

*^‡^SIVD vs. HC, *P* < 0.05.*

*^†^AD vs. HC, *P* < 0.05.*

### Association Between Mean Flow Velocity/Pulsatility Index and Cognitive Function in Patients With Dementia

**FIGURE 2:**
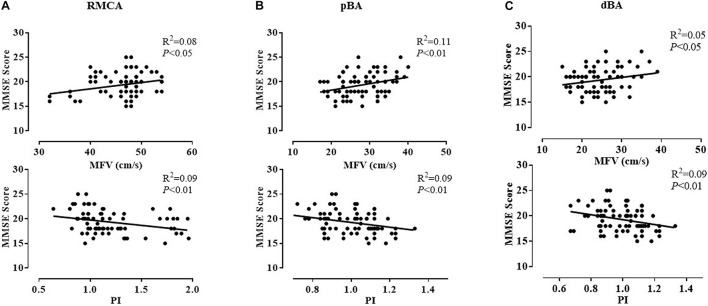
Correlation between MMSE scores and the MFV and PI of three representative arteries in patients with dementia. After adjusting for age, sex, and vascular risk factors, the MMSE score was positively correlated with the MFV of the right MCA (*P* < 0.05) **(A)**, pBA (*P* < 0.01) **(B)**, and dBA (*P* < 0.05) **(C)** and negatively correlated with the PI of the right MCA (*P* < 0.01) **(A)**, pBA (*P* < 0.01) **(B)**, and dBA (*P* < 0.01) **(C)**. dBA, distal basilar artery; MCA, middle cerebral artery; MFV, mean flow velocity; MMSE, Mini-Mental State Examination; pBA, proximal basilar artery; PI, pulsatility index.

Among all TCD parameters, the MMSE score was positively correlated with the MFV of the bilateral ICAs, MCAs, and PCAs; right ACA and VA; and pBA and dBA. It was negatively correlated with the PI of the bilateral MCAs; right ICA and VA; and pBA and dBA in all patients with either SIVD or AD. Sample plots for the MFV and PI in the right MCA as well as pBA and dBA from the linear regression analysis are shown in Figure 2. In terms of cognitive domains, the PI of the left MCA and PCA was negatively correlated with the memory composite score, and the MFV of the left ICA was positively correlated with the executive composite score. These correlations remained statistically significant after adjusting for age, sex, hypercholesterolemia, diabetes mellitus, coronary heart disease, hypertension, history of stroke, and BMI (Supplementary Tables 1–3).

### Association Between Carotid Atherosclerosis and Cognitive Function in Patients With Dementia

Among all carotid ultrasound parameters, number of arteries with plaque, total carotid plaque score, and right CCA lumen diameter were negatively correlated with the MMSE score in all patients (Supplementary Table 4). Total carotid plaque score and right CCA lumen diameter were negatively correlated with either memory composite or executive composite scores (Supplementary Table 5). After adjusting for age, sex, hypercholesterolemia, diabetes mellitus, coronary heart disease, hypertension, history of stroke, and BMI, these correlations were still statistically significant.

## Discussion

In this study, TCD revealed significantly decreased MFV and increased PI in most major cerebral arteries in patients with SIVD compared with those in HCs. We also found increases in lumen diameter, number of arteries with plaque, and total carotid plaque score in patients with SIVD as compared to the HC group. Patients with AD also showed intracranial hemodynamic disruption without prominent carotid atherosclerosis. Furthermore, the changes in intracranial hemodynamics and carotid atherosclerosis were correlated with cognitive impairment in dementia patients with either SIVD or AD. These correlations were independent of age, sex, hypercholesterolemia, diabetes mellitus, coronary heart disease, hypertension, history of stroke, and BMI. These findings suggest that hemodynamic and structural changes in cerebral and carotid large blood vessels are involved in the pathogenesis of SIVD and to a lesser extent AD.

Although a study did not observe any robust difference in MFV or PI measured with TCD between patients with dementia and HCs (Malojcic et al., 2017), a meta-analysis concluded that the MFV of the MCA was decreased in patients with both VaD and AD (Sabayan et al., 2012). Consistently, we found decreased MFV and increased PI in arteries of both anterior and posterior circulations in patients with SIVD and AD. Moreover, we found lower MFV of the left PCA and higher PI of the bilateral PCAs in SIVD compared to AD. Our results were consistent with a previous study showing higher PI and lower MFV in patients with small vessel disease compared to those with AD (Sattel et al., 1996).

Our abovementioned findings of intracranial hemodynamics and carotid atherosclerosis in patients with SIVD and AD were potentially influenced by the differences in demographic and clinical features between groups. In this study, fewer subjects had vascular risk factors in the AD group compared to the HC group (hypercholesterolemia 30 vs. 60%, hypertension 26.7 vs. 46.7%, and diabetes mellitus 3.3 vs. 20.0%). Since we had strict MRI criteria (including variant cerebrovascular lesions) for both SIVD and AD, patients with significant vascular risk factors may have been excluded from the AD group. However, patients with AD still demonstrated worse intracranial hemodynamics than HCs, suggesting possible independence between cerebrovascular disruption in AD pathophysiology and vascular risk factors. Moreover, the prevalence of vascular risk factors is higher in men than in women. Accordingly, dementia patients with more cardiovascular risk factors or male sex are likely to be diagnosed with VaD or mixed dementia in clinical practice (Jiao et al., 2021). It is therefore reasonable that male patients accounted for 59% of the SIVD group, which was more than 30% in the AD group. Furthermore, the difference in sex proportion between the SIVD and AD groups was also caused by the epidemiological features of both diseases (Podcasy and Epperson, 2016); specifically, more women suffer from AD than men (Babapour and van der Flier, 2019). Although it was previously reported that women had higher MFVs of the ACA, MCA, and PCA than men in a healthy population (Krejza et al., 1999), this sex-related difference in TCD parameters has not been widely demonstrated in other studies, especially in older populations (Patel et al., 2016).

Decreased MFV is commonly caused by arterial stenosis and cerebral blood flow reduction. Elevated PI indicates an increase in distal vascular resistance, which could lead to a decrease in blood flow velocity and cerebral metabolism. Taken together, the hemodynamic changes detected by TCD in our study revealed a whole-brain model of increasing resistance to blood flow and subsequent hypoperfusion in patients with SIVD and AD. Cerebral hypoperfusion was found to be associated with a series of pathophysiological processes, including amyloid deposition, aberrant tau hyperphosphorylation, cholinergic dysregulation, and neurovascular unit disruption. Increasing evidence indicates a critical role of cerebral hypoperfusion in the pathogenesis and progression of VaD and neurodegenerative diseases, including AD (Fleisher et al., 2009; Iadecola, 2017; Caruso et al., 2019; Moretti and Caruso, 2020).

Previous studies suggested a correlation between carotid atherosclerosis and MRI features of SIVD (Poels et al., 2012; Moroni et al., 2016; Zhai et al., 2020). In a community-based study, patients with more carotid plaques showed an increased risk of concomitant microatheroma in perforator arterials, which potentially led to ischemic parenchymal lesions such as lacunes and WMH (Zhai et al., 2020). An enlarged CCA diameter measured by TCD was associated with WMH (Rundek et al., 2017) and lacunar infarction (Brisset et al., 2013) in stroke-free participants, and it was also associated with microbleeds and PVS (Zhai et al., 2020). These studies support our findings of the pathophysiological carotid artery changes in patients with SIVD, such as increased carotid plaques and enlarged lumen diameter, which might be the compensatory mechanisms to maintain cerebral perfusion against increased arterial wall stiffness and thickness, and were closely related to increased intima-media thickness and plaque burden (Bonithon-Kopp et al., 1996). Consistent with a prospective cardiovascular health study, showing that carotid atherosclerosis did not significantly correlate with the increased risk of AD (Newman et al., 2005), we did not find notable carotid atherosclerosis in patients with AD. However, this result was potentially caused by the lower burden of vascular risk factors in patients with AD in this study, as compared to the HC group.

Interestingly, the cognitive function measured by the MMSE was positively correlated with the MFV and was negatively correlated with the PI of most major cerebral arteries in dementia patients with SIVD or AD. A linear positive correlation between cognitive impairment and the MFV of the MCA in patients with severe unilateral internal carotid artery stenosis was previously reported (Marshall et al., 2020). Moreover, higher MFV was associated with higher MMSE score and larger hippocampal volume in a population-based study (Ruitenberg et al., 2005). The longitudinal studies reported that the increased PI of either ACA or PCA predicted conversion to dementia in individuals with subjective memory decline or MCI (Chung et al., 2017) and the aggravation of cognitive decline in patients with mild-to-moderate AD (Lim et al., 2017). Apart from the global cognition, we also observed a negative correlation between memory and the PIs of the left MCA and PCA and a positive correlation between executive function and the MFV of the left ICA. These findings were supported by a previous study in which the PI of the MCA was correlated with information processing speed and executive function in patients with lacunar infarcts, even after the adjustment for vascular risk factors (Altmann et al., 2016).

Carotid atherosclerosis at early-to-mid stages characterized by intima-media thickening and plaques was associated with cognitive impairment in patients with dementia or MCI (Buratti et al., 2015; Gardener et al., 2017; Ihle-Hansen et al., 2019). Although the correlation between carotid artery lumen diameter and cognitive impairment has not been investigated in patients with cerebral vascular disease or AD, previous studies observed that the increased cerebral artery diameter that was measured by the time-of-flight MR angiogram was correlated with worse performance in memory and cognitive function in a population-based cohort of self-reported stroke-free individuals. In particular, participants with a larger left PCA diameter showed more declines in the scores of episodic memory, semantic memory, processing speed, and executive function in the 5-year follow-up (Gutierrez et al., 2018). Moreover, the cerebral arterial dilatation was related to AD pathology and was negatively correlated with the MMSE score in stroke-free participants (Gutierrez et al., 2019). Our findings provide new evidence of a correlation between carotid atherosclerosis (indicated by carotid plaque and lumen diameter) and cognitive function in dementia patients with either SIVD or AD, and this was independent of vascular risk factors.

We comprehensively investigated cerebral hemodynamics and carotid atherosclerosis using ultrasound technology and analyzed the correlations with cognitive impairment. However, this study was limited by the following factors. First, medical conditions associated with SIVD (e.g., metabolic syndrome and COPD) and statin use can potentially impact cerebral hemodynamics (Park et al., 2008; Giannopoulos et al., 2012; Hlavati et al., 2019). However, we did not analyze which factor most prominently contributed to hemodynamic changes. Second, since we only conducted the resting-state TCD measurement without end-tidal CO_2_ or acetazolamide enhancement, it is necessary to investigate cerebrovascular reactivities in patients with SIVD in future studies. Finally, to confirm a causal relationship between cerebral hemodynamics and cognitive impairment, it will be important to perform repeated blood flow measurements and cognitive assessments in longitudinal studies.

## Conclusion

In this study, we performed a non-invasive ultrasound and found prominent intracranial hemodynamic disruption and carotid atherosclerosis in patients with SIVD. The functional and pathological changes in cerebral and carotid large vessels were associated with cognitive impairment in dementia patients with SIVD or AD.

## Data Availability Statement

The original contributions presented in the study are included in the article/Supplementary Material, further inquiries can be directed to the corresponding author.

## Ethics Statement

The studies involving human participants were reviewed and approved by the Ethics Committee of Tianjin Medical University General Hospital (IRB2017-063-01). The patients/participants provided their written informed consent to participate in this study.

## Author Contributions

X-JL and NZ drafted the manuscript. X-JL, PC, MX, and X-BT collected and organized the data. X-JL and PC performed the statistical analyses. CG and XL performed the ultrasound measurements. NZ designed the study. All authors contributed to the article and approved the submitted version.

## Conflict of Interest

The authors declare that the research was conducted in the absence of any commercial or financial relationships that could be construed as a potential conflict of interest.

## Publisher’s Note

All claims expressed in this article are solely those of the authors and do not necessarily represent those of their affiliated organizations, or those of the publisher, the editors and the reviewers. Any product that may be evaluated in this article, or claim that may be made by its manufacturer, is not guaranteed or endorsed by the publisher.
