# Supplementary material for: Cerebral Hemodynamics and Carotid Atherosclerosis in Patients With Subcortical Ischemic Vascular Dementia
Source: Front Aging Neurosci. 2021 Nov 22;13:741881. doi: 10.3389/fnagi.2021.741881 (PMC8645960; doi:10.3389/fnagi.2021.741881)
Supplement: Supplementary file 1 [file Table_1.DOCX]

**Table 1 The association between MFV of insonated arteries and** **MMSE score in patients with dementia**

|  |  | Model 1 |  |  | Model 2 |  |  | Model 3 |  | |
| --- | --- | --- | --- | --- | --- | --- | --- | --- | --- | --- |
|  | β | 95% CI | *P* | β | 95% CI | *P* | β | 95% CI | | *P* |
| RICA | -0.07 | 0.89-0.98 | < 0.01 | -0.07 | 0.89-0.98 | < 0.01 | -0.06 | 0.89-0.98 | < 0.01 | |
| RACA | -0.08 | 0.87-0.97 | < 0.01 | -0.08 | 0.87-0.97 | < 0.01 | -0.08 | 0.87-0.97 | < 0.01 | |
| RMCA | -0.32 | 0.63-0.85 | < 0.01 | -0.31 | 0.64-0.86 | < 0.01 | -0.30 | 0.62-0.85 | < 0.01 | |
| RPCA | -0.12 | 0.82-0.95 | < 0.01 | -0.14 | 0.80-0.94 | < 0.01 | -0.14 | 0.80-0.95 | < 0.01 | |
| RVA | -0.84 | 0.27-0.70 | < 0.01 | -0.84 | 0.28-0.71 | < 0.01 | -0.87 | 0.26-0.70 | < 0.01 | |
| dBA | -0.29 | 0.68-0.84 | < 0.01 | -0.34 | 0.62-0.81 | < 0.01 | -0.36 | 0.61-0.81 | < 0.01 | |
| pBA | -0.28 | 0.68-0.84 | < 0.01 | -0.33 | 0.63-0.82 | < 0.01 | -0.36 | 0.61-0.81 | < 0.01 | |
| LICA | -0.06 | 0.91-0.99 | < 0.05 | -0.07 | 0.89-0.98 | < 0.01 | -0.08 | 0.88-0.98 | < 0.01 | |
| LMCA | -0.07 | 0.88-0.99 | < 0.05 | -0.11 | 0.84-0.96 | < 0.01 | -0.11 | 0.83-0.97 | < 0.01 | |
| LPCA | -0.11 | 0.85-0.95 | < 0.01 | -0.11 | 0.84-0.95 | < 0.01 | -0.11 | 0.85-0.97 | < 0.01 | |

Model 1 = Adjustments for sex, diabetes mellitus, hypertension, coronary heart disease, history of stroke and BMI. Model 2 = Model 1 + hypercholesterolemia. Model 3 = Model 2 + age. Among all TCD parameters, the MMSE score was positively correlated with MFV of RICA, RACA, RMCA, RPCA, RVA, pBA, dBA, LICA, LMCA and LPCA in all patients with either SIVD or AD. After adjusting for age, sex, hypercholesterolemia, diabetes mellitus, coronary heart disease, hypertension, history of stroke and BMI, the correlations were still statistically significant. There were no significant correlations between MMSE score and MFV of LACA and LVA. ACA, anterior cerebral artery; AD, Alzheimer’s disease; BMI, body mass index; dBA, distal basilar artery; ICA, intracranial internal carotid artery; L, left; MFV, mean flow velocity; MMSE, Mini-Mental State Examination; MCA, middle cerebral artery; PCA, posterior cerebral artery; pBA, proximal basilar artery; R, right; SIVD, subcortical ischemic vascular dementia; TCD, transcranial Doppler; VA, vertebral artery.

**Table 2 The association between PI of insonated arteries and MMSE score in patients with dementia**

|  |  | Model 1 |  |  | Model 2 |  |  | Model 3 |  |
| --- | --- | --- | --- | --- | --- | --- | --- | --- | --- |
|  | β | 95% CI | *P* | β | 95% CI | *P* | β | 95% CI | *P* |
| RICA | 4.08 | 1.28-2.58 | < 0.05 | 4.50 | 1.94-4.35 | < 0.05 | 4.50 | 1.95-4.35 | < 0.05 |
| RMCA | 9.06 | 1.24-1.96 | < 0.01 | 9.06 | 1.74-1.95 | < 0.01 | 8.52 | 1.21-3.72 | < 0.01 |
| RVA | 5.33 | 2.65-5.54 | < 0.05 | 6.32 | 5.93-6.34 | < 0.01 | 6.34 | 5.93-6.34 | < 0.01 |
| pBA | 12.00 | 1.29-2.53 | < 0.01 | 12.01 | 1.23-2.57 | < 0.01 | 12.51 | 1.28-2.59 | < 0.01 |
| dBA | 11.23 | 0.15-1.13 | < 0.01 | 11.23 | 0.35-1.85 | < 0.01 | 11.43 | 0.36-1.17 | < 0.01 |
| LMCA | 7.14 | 0.23-1.62 | < 0.01 | 8.57 | 0.23-1.24 | < 0.01 | 10.07 | 0.50-1.39 | < 0.01 |

Model 1 = Adjustments for sex, diabetes mellitus, hypertension, coronary heart disease, history of stroke and BMI. Model 2 = Model 1 + hypercholesterolemia. Model 3 = Model 2 + age. Among all TCD parameters, the MMSE score was negatively correlated with PI of RICA, RMCA, RVA, pBA, dBA, and LMCA in all patients with either SIVD or AD. After adjusting for age, sex, hypercholesterolemia, diabetes mellitus, coronary heart disease, hypertension, history of stroke and BMI, the correlations were still statistically significant. There were no significant correlations between MMSE score and PI of RACA, RPCA, LICA, LACA, LPCA and LVA. ACA, anterior cerebral artery; AD, Alzheimer’s disease; BMI, body mass index; dBA, distal basilar artery; ICA, intracranial internal carotid artery; L, left; MMSE, Mini-Mental State Examination; MCA, middle cerebral artery; PI, pulsatility index; PCA, posterior cerebral artery; pBA, proximal basilar artery; R, right; SIVD, subcortical ischemic vascular dementia; TCD, transcranial Doppler; VA, vertebral artery.

|  |  | Model 1 |  |  | Model 2 |  |  | Model 3 |  |
| --- | --- | --- | --- | --- | --- | --- | --- | --- | --- |
|  | β | 95% CI | *P* | β | 95% CI | *P* | β | 95% CI | *P* |
| Memory |  |  |  |  |  |  |  |  |  |
| LMCA-PI | 1.26 | 1.03-1.66 | < 0.05 | 1.27 | 1.06-1.56 | < 0.05 | 1.28 | 1.07-1.96 | < 0.05 |
| LPCA-PI | 1.18 | 1.05-1.74 | < 0.05 | 1.13 | 1.05-1.72 | < 0.05 | 1.29 | 1.04-1.83 | < 0.05 |
| Executive function |  |  |  |  |  |  |  |  |  |
| LICA-MFV | -0.39 | 1.08-1.82 | < 0.05 | -0.69 | 1.01-1.63 | < 0.05 | -0.34 | 1.06-1.87 | < 0.05 |

**Table 3 The association between TCD parameters and Memory/Executive function in patients with dementia**

Model 1 = Adjustments for sex, diabetes mellitus, hypertension, coronary heart disease, history of stroke and body mass index. Model 2 = Model 1 + hypercholesterolemia. Model 3 = Model 2 + age. Memory and executive function were presented and analyzed using composite Z scores. Memory composite score was negatively correlated with PI of LMCA and LPCA. Executive composite score was positively with MFV of LICA. After adjusting for age, sex, hypercholesterolemia, diabetes mellitus, coronary heart disease, hypertension, history of stroke and BMI, the correlations were still statistically significant. The other parameters of hemodynamics and carotid atherosclerosis that were not presented in this table did not show statistically significant correlation with either memory or executive function. BMI, body mass index; LMCA, left middle cerebral artery; LPCA, left posterior cerebral artery; LICA, left intracranial internal carotid artery; MFV, mean flow velocity; PI, pulsatility index; TCD, transcranial Doppler.

|  |  | Model 1 |  |  | Model 2 |  |  | Model 3 |  |
| --- | --- | --- | --- | --- | --- | --- | --- | --- | --- |
|  | β | 95% CI | *P* | β | 95% CI | *P* | β | 95% CI | *P* |
| Number of arteries with plaque | 0.44 | 1.05-2.31 | < 0.05 | 0.48 | 1.07-2.45 | < 0.05 | 0.48 | 1.07-2.46 | < 0.05 |
| Total carotid plaque score | 0.32 | 1.04-1.84 | < 0.05 | 0.31 | 1.02-1.84 | < 0.05 | 0.32 | 1.02-1.86 | < 0.05 |
| RCCA Lumen diameter | 0.23 | 1.04-1.58 | < 0.05 | 0.24 | 1.03-1.56 | < 0.05 | 0.22 | 1.02-1.62 | < 0.05 |

**Table 4 The association between carotid atherosclerosis and MMSE score in patients with dementia**

Model 1 = Adjustments for sex, diabetes mellitus, hypertension, coronary heart disease, history of stroke and BMI. Model 2 = Model 1 + hypercholesterolemia. Model 3 = Model 2 + age. Number of arteries with plaque, total carotid plaque score and lumen diameter of RCCA were negatively correlated with the MMSE score in all patients with either SIVD or AD. After adjusting for age, sex, hypercholesterolemia, diabetes mellitus, coronary heart disease, hypertension, history of stroke and BMI, the correlations were still statistically significant. There were no significant correlations between MMSE score and lumen diameter of other carotid arteries and plaque numbers. AD, Alzheimer’s disease; BMI, body mass index; MMSE, Mini-Mental State Examination; RCCA, right common carotid artery; SIVD, subcortical ischemic vascular dementia.

|  |  | Model 1 |  |  | Model 2 |  |  | Model 3 |  |
| --- | --- | --- | --- | --- | --- | --- | --- | --- | --- |
|  | β | 95% CI | *P* | β | 95% CI | *P* | β | 95% CI | *P* |
| Memory |  |  |  |  |  |  |  |  |  |
| Total carotid plaque score | 0.49 | 1.12-2.04 | < 0.05 | 0.74 | 1.03-1.61 | < 0.05 | 0.48 | 1.02-1.75 | < 0.05 |
| RCCA-Lumen diameter | 0.29 | 1.15-1.98 | < 0.01 | 0.92 | 1.02-1.53 | < 0.01 | 0.52 | 1.03-1.81 | < 0.05 |
| Executive function |  |  |  |  |  |  |  |  |  |
| Total carotid plaque score | 0.64 | 1.06-1.85 | < 0.05 | 0.73 | 1.12-1.98 | < 0.05 | 0.45 | 1.05-1.92 | < 0.05 |
| RCCA-Lumen diameter | 0.71 | 1.07-1.93 | < 0.01 | 0.86 | 1.13-2.23 | < 0.01 | 0.58 | 1.02-1.79 | < 0.01 |

**Table 5 The association between carotid atherosclerosis and Memory/Executive function in patients with dementia**

Model 1 = Adjustments for sex, diabetes mellitus, hypertension, coronary heart disease, history of stroke and BMI. Model 2 = Model 1 + hypercholesterolemia. Model 3 = Model 2 + age. Memory and executive function were presented and analyzed using composite Z scores. Memory composite score was negatively correlated with total carotid plaque score and lumen diameter of RCCA. Executive composite score was negatively with total carotid plaque score and lumen diameter of RCCA. After adjusting for age, sex, hypercholesterolemia, diabetes mellitus, coronary heart disease, hypertension, history of stroke and BMI, the correlations were still statistically significant. The other parameters of hemodynamics and carotid atherosclerosis that were not presented in this table did not show statistically significant correlation with either memory or executive function. BMI, body mass index; RCCA, right common carotid artery.
